# Prevalence and sociodemographic patterns of physical activity among Bangladeshi young adults

**DOI:** 10.1186/s41043-017-0108-y

**Published:** 2017-07-14

**Authors:** Riaz Uddin, Asaduzzaman Khan, Nicola W Burton

**Affiliations:** 1 Department of Pharmacy, Stamford University Bangladesh, Dhaka, 1217 Bangladesh; 2 School of Health and Rehabilitation Sciences, The University of Queensland, St Lucia, Brisbane, QLD 4072 Australia; 3 School of Human Movement and Nutrition Sciences, The University of Queensland, St Lucia, Brisbane, QLD 4072 Australia

**Keywords:** Exercise, Early adulthood, Low- and middle-income country, Bangladesh, South Asia

## Abstract

**Background:**

Physical activity offers physical and psychosocial health benefits that are important during young adulthood and later in life. However, little is known about the physical activity of young adults in low- and middle-income countries. The purpose of this study was to estimate the participation of physical activity in Bangladeshi young adults and to assess differences by gender, age and family income.

**Methods:**

This cross-sectional study with a self-administered survey used a convenience sample of 573 young adults aged 18–24 years from six purposively selected universities in Dhaka City, Bangladesh. Data were collected during September–November 2015. Medians and their interquartile ranges of weekly time spent in total physical activity, and in different domains of physical activity, were computed. Non-parametric equality of medians test was used to examine gender differences in the median values. Chi-square test and Fisher’s exact test were used to examine gender differences in the prevalence of meeting physical activity recommendations and frequency of participation in different leisure-time physical activities, and differences in meeting the activity recommendations by age and family income.

**Results:**

Seventeen percent of the participants were meeting moderate-to-vigorous physical activity (MVPA) recommendations with a significantly higher proportion of males than females (27 vs. 6%, *p* < .0001). Median duration of MVPA was significantly higher (*p* < .0001) for males [120 min/week (80, 190)] than females [90 min/week (50, 120)]. Jogging/running was the most commonly reported leisure-time physical activity, with 20% of males and 12% of females doing this at least once a week. Age and family income were not significantly associated with meeting MVPA recommendations.

**Conclusions:**

Four out of five young adults in Dhaka City did not meet the physical activity recommendations. Additional population-based studies, including regional and metropolitan areas, and using objective measurement, are needed to understand the physical activity patterns of Bangladeshi young adults.

## Introduction

Regular physical activity participation offers physical and psychosocial health benefits including physical fitness, healthy weight, and prevention and management of conditions such as diabetes and cardiovascular disease, stress and depression [1]. Despite these benefits, a large number of people worldwide are leading an inactive lifestyle. According to a global estimation in 2010 by the World Health Organisation (WHO), approximately 23% of adults aged 18 years or more did not meet the WHO recommendations of at least 150 min/week of moderate-intensity or 75 min/week vigorous-intensity activity, or an equivalent combination of moderate-to-vigorous physical activity [2]. In the WHO South-East Asian region, 15% of adults were not meeting the WHO recommended levels of physical activity [2].

According to the WHO, physical inactivity is the fourth leading risk factor for global mortality [3, 4], and evidence suggests that inactivity-related deaths have increased over the years [5]. In 2008, 3.2 million of global deaths per annum were attributable to physical inactivity [4], which increased to 5.3 million global deaths per annum as per the most recent estimate in 2012 [6]. Insufficient levels of physical activity have significant implications for the public health burden associated with non-communicable diseases. Non-communicable diseases are the leading cause of death worldwide. Globally, deaths attributable to these diseases are rising at an alarming rate. Low- and middle-income countries are particularly vulnerable to non-communicable disease-related mortality and morbidity [7]. In 2004, it was projected that non-communicable disease-related mortality in the South-East Asian region would increase by approximately 60% by 2030 [8]. In 2008, non-communicable diseases accounted for an estimated 55% of total deaths in this region [8] and are likely to exceed 75% by 2030 [9]. Though currently there are no overall regional data available, non-communicable disease-related morbidity shows a steady increase in the South Asian countries [8]. Given the considerable increase in the burden of non-communicable disease in South Asian countries, and the protective role of physical activity against non-communicable diseases [1], it is imperative to better understand the physical activity profile of people living in this region.

Young adulthood, from age 18 to 24 years [10], is the transition from adolescence and an important phase of life [11]. It involves significant life events; young adults may move away from home gaining full residential independence, commence work or tertiary education and have more freedom in making lifestyle choices [10, 12]. During this transition, young adults may adopt unhealthy lifestyle choices and engage in low levels of physical activity [13]. Though physical activity participation has been observed to decline across most of the lifespan, late adolescence and young adulthood years can have the most significant decrease in physical activity [14, 15]. Physical inactivity during young adulthood can increase the risk for non-communicable diseases later in life [16]. Regular physical activity lowers cardio-metabolic risk factors of non-communicable diseases [17], reduces weight gain and increases the likelihood of weight loss and weight maintenance during young adulthood [18]. Young adults engaging in regular physical activity have been reported to have higher self-esteem, a more positive body image and more positive health perceptions than their inactive counterparts [19]. It has also been observed that young adults participating in high levels of physical activity have significantly lower levels of anxiety and depression [20] and better mental health and satisfaction with life than inactive young adults [21]. Thus, physical activity provides immediate benefits for young adults such as improved psychological well-being and cognitive performance [22].

Young adults’ physical activity behaviour has predominantly been studied in high-income countries, and little is known about low- and middle-income countries. Available evidence, mostly from university students, suggests that the prevalence of physical inactivity in young adults in low- and middle-income countries is higher (44%) than in high-income countries (39%) [23]. The few studies in Asian countries have reported the prevalence of inactivity among young adults to be 41% in Malaysia [24], 50% in Taiwan, 52% in South Korea, 71% in China and 75% in Japan [25]. Although some physical activity data for Bangladesh are available for adults aged ≥25 years [23, 26], no data are available for young adults. Bangladesh is a South Asian country and is ranked as the ninth most populated country in the world with a population density of 1298 people per square kilometre as of July 2015 [27]. Given the established relationships between inactivity and non-communicable diseases [28], and the significant increase in non-communicable diseases in Bangladesh in recent years [29], it is crucial to collect physical activity prevalence data from Bangladeshi young adults.

Available evidence suggests that males are more active than females in low- and middle-income countries [23, 24, 30] as well as in Asian countries [24, 25]. However, there is little evidence for physical activity variation by other socio-demographic factors for young adults in this region. Some research in low- and middle-income and Asian countries has found physical activity levels decrease with age [23] and family income in this population group [24]. To our knowledge, no study has offered a comprehensive overview of the sociodemographic pattern of physical activity participation of young adults in Bangladesh.

Hence, the present study aimed to assess the prevalence of participation of physical activity in young adults in Bangladesh and explore differences by age, gender and family income. This information can inform interventions to increase opportunities for and engagement in physical activity of Bangladeshi young adults residing in the metropolitan areas of the country.

## Methods

### Study participants

This cross-sectional study with a self-administered survey was conducted during September–November 2015 in a convenience sample of young adults aged 18–24 years from six purposively selected universities (three public and three private) in Dhaka City, the capital of Bangladesh. In order to estimate the prevalence of young adults’ physical activity with a 5% margin of error and 95% confidence interval, it was determined that this study needed at least 345 participants to achieve a power of 80% with the proportion of physically active students as 66% from an earlier study [31]. From a list of 49 public/private universities, which offer undergraduate programmes in Dhaka [32], eight were chosen based on their size, diversity of students, convenience and connection with the primary author. Of the eight invited to participate in this study, six universities (public = three and private = three) agreed. After obtaining approvals from the university authority, the principal investigator (first author) consulted with the institution nominated representatives (e.g. class lecturer) for a suitable time to access the students during class time. The principal investigator explained the context of the study emphasising the voluntary nature of study participation and then verbally invited the students to participate. Participants were recruited based on the following inclusion criteria: (1) undergraduate student, (2) aged 18 to 24 years and (3) permanent residents of Bangladesh. Written informed consent was obtained from all participants. In Bangladesh, the medium of instruction is English at university undergraduate level. Thus, the written survey was completed in English: this took approximately 40–45 min.

### Physical activity assessment

The Global Physical Activity Questionnaire (GPAQ) was developed by the WHO for population surveillance of physical activity in low- and middle-income countries [33, 34] and has been used in more than 100 countries through the STEPwise Approach to Non-communicable Disease Risk-Factor Surveillance (STEPS) programme [34]. Reliability and validity of the GPAQ has been assessed among adults aged ≥18 years in nine countries, including Bangladesh [33, 34], and found to have reproducible data and a ‘moderate’ to ‘strong’ positive correlation with the International Physical Activity Questionnaire (IPAQ). The current study used the GPAQ self-administered version, which is a relatively inexpensive method with comparable reliability and validity to the interview administration [35].

The GPAQ consists of 16 items on physical activity in three domains: work, commuting (travel to and from places) and during leisure time in a typical week [34]. Items ask about moderate and vigorous intensity for the work and leisure domains and moderate-intensity activity for the transport domain (walking or bicycling). Moderate-intensity activities are defined as ‘activities that require moderate physical effort and cause small increases in breathing or heart rate’. Vigorous-intensity physical activities are defined as ‘activities that require hard physical effort and cause large increases in breathing or heart rate’ [34]. The questionnaire contained a number of visual illustrations with examples of different physical activities to explain the concepts of moderate- and vigorous-intensity physical activity. For each domain, the participants were asked about the number of days physical activity was done in a typical week and the hours/minutes spent doing such activities in a typical day.

The survey data were cleaned based on the GPAQ protocol [36]. The following criteria were used to identify invalid responses:

- If the value for activity was more than 16 h/day in any of the physical activity sub-domains (vigorous-intensity work, moderate-intensity work, transport, vigorous-intensity leisure, or moderate-intensity leisure activity)
- Improbable response such as activity reported for more than 7 days in a week;
- Inconsistency in answering (e.g. transport activity was done 0 days in a week, but reported >0 min in the hour column) [36]

Consistent with the GPAQ data analysis guideline, all activity data were converted to minutes and were multiplied by the corresponding number of days [36]. Vigorous activity minutes were weighted by two given the higher intensity than moderate activity [36]. The time spent in vigorous activity (weighted) and moderate activity minutes were summed to obtain a measure of the total minutes/week spent doing moderate-to-vigorous physical activity (MVPA). Participants were then categorised as meeting the WHO recommendations or not, using the criterion of ≥150 min/week of MVPA [36].

Additional items were used to assess participation in specific types of physical activity. Participants were asked to report total time spent walking for recreation, exercise, or to get to or from places during a typical weekday and weekend day. Participants also indicated the frequency of engaging in each of 18 specific types of leisure-time physical activity (such as jogging/running, cricket, football, swimming/water exercise, gym and yoga) with response options of daily, 2–3 times a week, at least once a week, and none.

### Other measures

Participants also completed survey items to assess: age, gender, marital status, height and weight, university type, year of study, parents’ educational qualification and monthly gross household income. Due to a relatively narrow range (18–24 years), age was grouped into two categories: 18–20 and 21–24 years. Body mass index (BMI) was computed from self-reported height and weight and then grouped into one of the three BMI categories based on criteria from the WHO: normal (<18.50 kg/m^2^); underweight (18.50–24.99 kg/m^2^) and overweight (≥25.00 kg/m^2^) [37]. Monthly gross household income was used as a proxy socio-economic status indicator as done elsewhere [38] and categorised into one of four groups (≤20,000 Bangladeshi currency-BDT; 20,001–40,000 BDT; 40,001–70,000 BDT; and >70,000 BDT).

A draft version of the questionnaire was piloted in a small convenience sample of undergraduate students of a university based in Dhaka City, Bangladesh (*n* = 30, male = 15, female = 15). The aim of this pilot was to evaluate the feasibility of administering the questionnaire. Feedback from the pilot participants indicated that the questionnaire could be easily understood and answered.

### Statistical analyses

The characteristics of the participants were summarised using descriptive statistics. Due to non-normal distribution of physical activity data, MVPA minutes/week were summarised using the median with interquartile ranges. Differences between physical activity medians by gender were analysed using non-parametric equality-of-medians test. The prevalence of meeting the WHO MVPA recommendations, and frequency of participation in different types of leisure-time physical activity were examined for possible gender differences and are reported as percentages with 95% confidence intervals for males and females separately. Using Chi-square test and Fisher’s exact test, physical activity prevalence was further examined for possible age and income differences. This was done separately for males and females, as physical activity prevalence is likely to differ by gender [23, 39]. Statistical significance was set at 5%. Data were analysed using STATA version 13 (StataCorp LP., College Station, Texas).

## Results

### Study participants

A total of 628 students were invited to participate in the study, and 575 completed the questionnaire (response rate 91.6%). Two respondents were excluded as they provided improbable or out of range data. As a result, the analytical sample of the study consisted of 573 students with 45% female and an average age of 20.7 years (SD = 1.35). Participants were predominantly single (94%), with the majority living with their parents (47%). Details on the socio-demographic characteristics of the study participants are presented in Table 1.

**Table 1:** Characteristics of the participating young adults in Dhaka City, Bangladesh (*n* = 573)

Characteristics	Number^a^	Percentage (%)
Age (years)		
18–20	262	45.7
21–24	311	54.3
Gender		
Male	313	54.6
Female	260	45.4
Marital status		
Single	538	93.9
Married or others (e.g. de facto, divorced, separated)	35	6.1
BMI		
Normal range	353	61.7
Underweight	139	24.3
Overweight	80	14.0
University type		
Public	277	(48.3)
Private	296	(51.7)
Year of study at university		
First year	184	32.1
Second year	223	38.9
Third year	166	29.0
Mother’s educational qualification		
Primary or equivalent	111	19.4
Secondary (or equivalent)	147	25.7
Higher secondary (or equivalent)	125	21.9
Tertiary (or equivalent)	188	32.9
Father’s educational qualification		
Primary or equivalent	53	9.3
Secondary (or equivalent)	64	11.2
Higher secondary (or equivalent)	102	17.9
Tertiary (or equivalent)	352	61.7
Monthly gross family income (in BDT)^b^		
≤20,000	115	20.3
20,001–40,000	162	28.6
40,001–70,000	172	30.4
>70,000	117	20.7

^a^Total for each variable may not be equal to *n* = 573 due to missing values

^b^BDT = Bangladeshi Taka (local currency); 1,000 DBT = 12.37 USD as of 6 July 2017

### Physical activity participation

Of the 573 participants, 17% (95% CI 14–21%) met MVPA recommendations to do ≥150 min/week with significantly (*p* < .0001) more males [27% (22–32%)] than females [6% (4–10%)] meeting the recommendations.

Although more participants in the older group met MVPA recommendations, age was not significantly associated with meeting the recommendations (Table 2). Among males, 23% of those aged 18–20 years and 30% of those aged 21–24 years met MVPA recommendations (*p* = .24). The proportions of females meeting the recommendations were 6% of those aged 18–20 and 7% of those aged 21–24 years (*p* = .65).

**Table 2:** Percentages (95% CIs) of male and female young adults in Dhaka City, Bangladesh, meeting the WHO physical activity recommendations by age group and family income

Variable	Male			Female			Overall		
	% active	95% CI	*p* value	% active	95% CI	*p* value	% active	95% CI	*p* value
Age (years)									
18–20	23.1	16.3–31.2	.24^a^	5.5	2.2–10.9	.65^a^	14.5	10.5–19.4	.11^a^
21–24	29.5	22.5–36.3		6.8	3.2–12.5		19.6	15.3–24.5	
Monthly family income (BDT)*									
≤20,000	25.0	16.4–35.4	.33^a^	3.7	0.1–1.9	.99^b^	20.0	13.1–28.5	.20^a^
20,001–40,000	33.3	23.9–43.9		5.8	1.6–14.2		21.6	15.5–28.7	
40,001–70,000	21.4	13.2–31.7		6.8	2.5–14.3		14.0	9.1–20.0	
> 70,000	26.7	14.6–41.9		6.9	2.3–15.5		14.5	8.7–22.2	

*p* < .05 was considered to be statistically significant

*BDT* Bangladeshi Taka, *CI* confidence interval

^a^Based on Chi-square test

^b^Based on Fisher’s exact test

*1,000 BDT = 12.37 USD as of 6 July 2017

The proportion of participants meeting MVPA recommendations across the four income groups ranged from 14 to 22% with no statistically significant differences (*p =* .20). The proportion of participants meeting recommendations tended to be higher in the lower two income groups than the two higher income groups. The proportion of males meeting the MVPA recommendations ranged from 21 to 33% across the four income groups without any significant linear trend (*p* = .33). The proportion of females meeting the recommendations increased with increasing family income, though there was no statistically significant association (4 to 7%, *p* = .99) (Table 2).

### Participation in different domains of physical activities

**Fig. 1:**
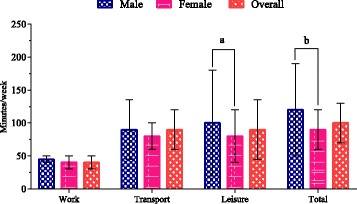
Distribution of physical activity (median minutes/week) in young adults in Dhaka City, Bangladesh, by gender. *a* Significant at *p* < .05. *b* Significant at *p* < .0001. *Error bars* represent interquartile ranges. Work domain represents 190 participants who reported doing at least 10-min bouts of work-related physical activity; male = 94, female = 96. Transport domain represents 305 participants who reported doing at least 10-min bouts of active transport; male = 183, female = 122. Leisure domain represents 291 participants who reported doing at least 10-min bouts of recreational physical activity; male = 199, female = 92. Total physical activity represents the overall survey population; male = 313, female = 260

Median time spent in total MVPA was 100 min/week (IQR 60, 130) with males spending significantly more time [120 min/week (IQR 80, 190)] than females [90 min/week (IQR 50, 120)] (*p* < .0001). Although there was a trend for males to spend more time than females in each of work, commuting and leisure-time physical activity, this difference was statistically significant only for leisure (Fig. 1). One-third (33%) of the participants (male 30%, female 37%) reported some form of work-related physical activity. Of these, the median time was 45 min/week (IQR 30, 50) for males and 40 min/week (IQR 15, 50) for females. Just over half of the participants (overall 53%, male 58% vs. female 47%) reported bicycling or walking for transport with a median of 90 min/week (IQR 60, 135) for males and 80 min/week (IQR 20, 120) for females. Half of the participants (overall 51%, male 64% vs. female 35%) engaged in leisure-time physical activity, with significantly more time among males [100 min/week (IQR 60, 180)] than females [80 min/week (IQR 60, 120)] (*p* = .03).

### Time spent in walking

Median duration of walking did not differ significantly between males and females. During a typical weekday, the median time spent walking was 30 min (IQR 30, 60) with slightly longer times among males [40 min (IQR 30, 60)] than females [30 min (30, 60)]. Median walking time during a typical weekend day was 30 min (IQR 20, 50) with a median of 30 min for both males (IQR 30, 60) and females (15, 40).

### Types of leisure-time physical activities

**Fig. 2:**
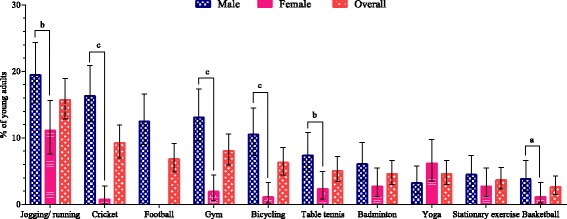
Young adults’ participation (percentages with 95% CIs) in different leisure time physical activities (selected top 10 leisure-time physical activities only) by gender, Dhaka City, Bangladesh. *a* Significant at *p* < .05. *b* Significant at *p* < .01. *c* Significant at *p* < .0001. *Error bars* represent 95% CIs. *CI* confidence interval

Figure 2 illustrates the top 10 types of leisure-time physical activity (excluding walking). Jogging/running was the most common type of leisure-time physical activity for the entire sample. Overall, 15.7% (95% CI 12.8–19.0%) participants reported jogging/running at least once a week, with 19.5% (15.3–24.3%) of males and 11.2% (7.6–15.6%) of females. Males participated more frequently in team sports, such as cricket, football etc. Cricket was the second most frequently reported leisure-time physical activity for males [16.3% (12.4–20.9%)]. Among males, 12.5% (9.0–16.6%) did football at least once a week, with no females playing football. More than one in ten [13.1% (9.6–17.4%)] males reported going to a gym at least once a week. Apart from jogging/running, females reported participating in indoor physical activities, such as yoga [6.2% (3.6–9.8%)], and exercise such as using a stair climber and treadmill [2.7% (1.1–5.5%)] and playing badminton [2.7% (1.1–5.5%)].

## Discussion

Physical inactivity may negatively affect current physical and psychosocial health of young adults and has long-term health consequences for non-communicable diseases. The present study found that four out of five young adults in Dhaka City, Bangladesh, did not meet the WHO recommendations of at least 150 min per week of MVPA with more females (94%) than males (73%) not meeting recommendations. The percentage of participants not meeting physical activity recommendations in our study was higher than in previous studies with other age groups in Bangladesh using the same survey instrument (GPAQ). Those studies found that 27 to 52% of the participants aged ≥25 years did not meet the recommendations [5, 26, 40, 41].

In our study, females were less active than males, which is consistent with previous studies across a range of countries including low- and middle-income and South Asian countries [23, 30, 42]. An earlier multi-country study found a significant gender difference (favouring males) in physical activity in young adults regardless of the country status [30]. In a recent study, a similar trend was observed in three South Asian Countries—Bangladesh, India and Pakistan [23]. This gender difference in physical activity may reflect the social norms and gender roles that are common in Bangladeshi culture. In Bangladesh, females tend to have restricted outdoor engagement in recreational and social activities that often starts from puberty [43]. Socio-cultural customs in Bangladeshi society can separate males and females into two different worlds [44]. While males can go outside, socialise and take part in outdoor activities such as cricket or football or cycling, females are often confined to domestic chores and not allowed to go outside freely [44, 45]. Females are often restricted from exposure to non-family males and participating in team sports due to social taboo [44]. Parents in Bangladesh may impose restrictions on female adolescents due to safety concerns [46]. For example, a study in Dhaka reported that 76% of female adolescents aged 14 to 16 years had experienced eve teasing (sexual/street harassment) [47]. Thus, parents are likely to restrict or discourage their daughters from outdoor activities such as recreational walking or bicycling. It has been argued that gender differences in physical activity participation might also reflect gender differences in preferences for activities, with females preferring more passive social activities and males preferring more physically demanding pursuits [48].

The clear gender difference in amount and types of physical activity observed in this study is crucial for physical activity promotion and policy implementation in the country. While promoting physical activity is important for all, female young adults are a priority group. More research is needed, therefore, to understand the factors that might positively and negatively influence physical activity participation of female young adults. Social factors such as family support; cognitive factors like enjoyment of physical activity and self-efficacy; and environmental factors such as perceived safety, availability, cost and access to physical activity facilities have previously been identified as particularly important for physical activity of female young adults [49].

According to the current study, work-related physical activity contributed the least to total MVPA for both males and females. This is understandable as the participants were university students and therefore unlikely to engage in physically demanding occupations. In Bangladesh, some undergraduate students may work by providing private tuition, which requires no manual labour. A third of the participants in this study reported doing some amount of work-related physical activity with a higher rate of participation for females (36%) than males (30%). This might reflect involvement in household activities, such as cleaning and home maintenance, cooking and shopping, which has been previously identified as more common among females than males in other low- and middle-income countries [50]. In Bangladeshi family culture, women often are responsible for taking care of the children and other family members, preparing the meals and doing other domestic tasks. Although the participants in this study were predominantly single (94%) and therefore perhaps free from family responsibilities, they may still do domestic activities if living with friends/classmates or alone. More research is needed to better understand how domestic activities contribute to physical activity of Bangladeshi young adults, in particular young women.

The current study indicated that just over half of the participants (male 58%, female 47%) reported bicycling or walking for transport. Several social and environmental factors might limit active commuting in Bangladesh. Rickshaws, a human-peddled small vehicle, are readily available and accessible to shuttle small distances rather than walking/bicycling. Lack of safe footpaths for walking and pathways for bicycling may be environmental barriers. Young females are vulnerable to crime while travelling and so are more likely to avoid walking [51]. In the metropolitan areas, such as in Dhaka, the streets are often not well lit at night and roads are highly congested with heavy traffic. Moreover, the social structure in Bangladesh does not support females bicycling to or from work as this is not a culturally acceptable norm. Transport-related physical activity could be promoted by building safe pathways for walking and cycling, ensuring the streets are well lit during night and thus improving perceived safety.

Other research has indicated that males prefer more physically demanding physical activity like team sports and females tend to participate in physical activity such as walking, dance, aerobics and yoga [49]. This is consistent with the findings of our study, where significantly more males engaged in outdoor leisure-time physical activity and team sports than females. Females more commonly reported doing yoga and indoor physical activity than males. However, female participation in jogging/running was comparable to the males, which seems contradictory. Other research, however, has also indicated that this is often an individual activity preferred by females [49].

The current study found no statistically significant association between age and physical activity participation, which is consistent with the findings of a recent study in Malaysia that used a similar age group [24]. However, previous studies in low- and middle-income countries suggest an inverse relationship between age and physical activity in young adults [23, 38]. Those studies, however, used a more aged varied sample, including adolescents aged 16 to 18 years, than the current study. It has previously been suggested that the greatest decline in physical activity occurs during the adolescent period (age 13–18 years) and slows during young adulthood [52]. More research is needed to understand the relationship between age and physical activity in young adults in low- and middle-income countries.

Consistent with the findings of a previous study in South Africa [38], family income was not associated with physical activity in this study. This may be because the participants in this study lived in areas where the logistics for physical activity did not differ by income. For example, the built environment for physical activity in Bangladesh does not vary greatly across residential areas, and available outdoor physical activity facilities, such as parks and playing fields, are accessible to everyone. In this study, there was, however, a trend for physical activity to be lower in the higher income groups. It is generally assumed that high-income enables access to paid physical activity facilities (e.g. gym, sports club) or home-based physical activity equipment [53]. However, as observed in adolescents from Thailand, which is another Asian country, it may be that high income enables more sedentary activities such as use of smartphones, computers, laptops and video gaming facilities [53]. Income might be a relatively less important influence on physical activity of young adults in Bangladesh than individual factors such as attitudes and motivation.

The study had some limitations that warrant consideration. It used a non-random convenience sample of university students from a metropolitan city. As such, the results of this study do not represent all young adults of the country and may have limited generalizability. The prevalence of physical activity presented in this paper was not adjusted for potential confounders; therefore, these results should be interpreted with caution. Physical activity was assessed using self-report, which is a common and convenient method in large population-based studies, but vulnerable to social desirability and recall bias. Data were collected during the pre-examination period for some universities, and so some students’ physical activity could have been negatively influenced by their academic commitments. Other items used in the survey to assess total walking time for weekdays and weekend days and frequency of doing different leisure-time physical activities were not validated.

## Conclusions

The present study found that four out of five young adults in Dhaka City, Bangladesh, did not meet the WHO recommended level of physical activity for optimal health, with higher rates of insufficient activity among females than males. As physical inactivity is a well-documented risk factor for adverse health outcomes including non-communicable diseases, the findings of this study are alarming. Additional population-based studies, preferably, longitudinal studies with representative samples from regional and metropolitan areas and objective measurement of physical activity, are needed to understand the factors associated with physical activity in Bangladeshi young adults, in particular among females.

## Abbreviations

BDT: Bangladeshi taka (local currency)
CI: Confidence interval
GPAQ: Global Physical Activity Questionnaire
IPAQ: International Physical Activity Questionnaire
IQR: Interquartile range
MVPA: Moderate-to-vigorous physical activity
SD: Standard deviation
STEPS: STEPwise Approach to Non-communicable Disease Risk-Factor Surveillance
USD: United States Dollars
WHO: World Health Organisation
